# The association between vitamin D intake with inflammatory and biochemical indices and mortality in critically ill patients with COVID‐19: A case‐control study

**DOI:** 10.1002/iid3.844

**Published:** 2023-04-26

**Authors:** Maryam Gholamalizadeh, Faezeh Rabbani, Mina Ahmadzadeh, Azadeh Hajipour, Hayehe Musavi, Khadijeh Abbasi Mobarakeh, Zahra Salimi, Bojlul Bahar, Zahra Mahmoodi, Somayeh Gholami, Samaneh Mirzaei Dahka, Saeid Doaei, Mokammad Esmail Akbari

**Affiliations:** ^1^ Cancer Research Center Shahid Beheshti University of Medical Sciences Tehran Iran; ^2^ Department of Medicinal Chemistry, Faculty of Pharmacy and Pharmaceutical Sciences Research Center Shahid Sadoughi University of Medical Sciences Yazd Iran; ^3^ Department of Clinical Nutrition and Dietetics, National Nutrition and Food Technology Research Institute, Faculty of Nutrition and Food Technology Shahid Beheshti University of Medical Sciences Tehran Iran; ^4^ School of Health Qazvin University of Medical Sciences Qazvin Iran; ^5^ Tabriz University of Medical Sciences Rasht Iran; ^6^ Department of Community Nutrition, Nutrition and Food Security Research Center, School of Nutrition and Food Science Isfahan University of Medical Sciences Isfahan Iran; ^7^ Nutrition and Metabolic Diseases Research Center Ahvaz Jundishapur University of Medical Sciences Ahvaz Iran; ^8^ Nutrition Sciences and Applied Food Safety Studies, Research Centre for Global Development, School of Sport & Health Sciences University of Central Lancashire Preston UK; ^9^ Department of Nutrition, Science and Research Branch Islamic Azad University Tehran Iran; ^10^ Razi Hospital, Guilan University of Medical Sciences Rasht Iran; ^11^ School of Nursing and Midwifery, Guilan University of Medical Sciences Rasht Iran; ^12^ Department of Community Nutrition, National Nutrition and Food Technology Research Institute, Faculty of Nutrition Sciences and Food Technology Shahid Beheshti University of Medical Sciences Tehran Iran

**Keywords:** Covid‐19, critical ill patientvitamin D, survivalcritical ill patient, vitamin D survival

## Abstract

**Background:**

The coronavirus disease‐2019 (COVID‐19) has become a worldwide health issue with widespread hospitalization and dependence on the intensive care unit (ICU). Vitamin D has a key role in modulating immune cells and modulating the inflammatory responses. This study aimed to investigate the association of vitamin D supplementation with inflammatory, biochemical, and mortality indices in critically ill patients with COVID‐19.

**Methods:**

This case‐control study was conducted on critically ill COVID‐19 patients hospitalized in the ICU including the survived >30 day patients as the case group and dead patients as the control group. The status of vitamin D supplementation and inflammatory and biochemical indices of the patients were retrieved from the medical records. Logistic regression method was used to assess the association between 30 days survival and vitamin D supplement intake.

**Results:**

Compared to the group of COVID‐19 patients who died in <30 day, the survived patients had a lower eosinophile level (2.2 ± 0.5 vs. 6 ± 0.0, *p* < .001) and higher vitamin D supplementation duration (9 ± 4.4 vs. 3.3 ± 1.9 day, *p* = .001). Vitamin D supplementation had a positive association with survival in COVID‐19 patients (OR: 1.98, 95% CI: 1.15−3.40, *p* < .05). The association remained significant after adjustments fot age, sex, underlying diseases, and smoking.

**Conclusion:**

Vitamin D supplementation in critically ill patients with COVID‐19 has the potential to increase survivability within the first 30 days of hospitalization.

## Graphical abstract

Vitamin D supplementation in critically ill patients with COVID‐19 have the potential to increase survivality within the first 30 days of hosptilization.

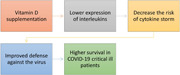

## 1 INTRODUCTION

Coronavirus disease 2019 (COVID‐19), a pandemic caused by severe acute respiratory syndrome coronavirus 2 (SARS‐CoV‐2), is the most challenging pandemic of the 21st century.^1^ The disease was first reported in Wuhan, China, and globally, as of November 2022; there have been 632 million confirmed cases and 6.6 million deaths, reported to World Health Organization. About 6 million people have been reported infected in Iran, and over 128,000 people have lost their lives.^2^ COVID‐19 patients have shown a wide range of symptoms including fever, cough, diarrhea, fatigue, nausea, and vomiting. More severe complications such as acute respiratory distress syndrome (ARDS) led to hospitalization and the urgent need for management in intensive care units (ICUs).^3^

Evidence suggested that the 4‐week rate of mortality of critically ill patients with COVID‐19 could be as high as 62%.^4^ Therefore, timely treatment of these patients based on reliable predictors of COVID‐19 severity, such as inflammatory and biochemical indices, is imperative.^5^ Studies have also demonstrated that predictors such as C‐reactive protein (CRP), interleukin 6 (IL‐6), tumor necrosis factor (TNF)‐α, neutrophil count, white blood cell (WBC) count, platelet count, and serum ferritin can provide valuable insight useful for assessing the progression of the disease to critical illness.^6^, ^7^, ^8^ At present, one of the most imperative ways to deal with this disease is to control risk factors. Vitamin D (vit D) deficiency is identified as one of the most conspicuous nutritional risk factors that can potentially influence the disease progression and mortality rate.^9^ Several studies reported that a lower serum level of vit D was associated with a higher COVID‐19 infection rate and worse outcomes in these patients, for instance, an increased mortality rate.^10^

Vit D is a steroid hormone endogenously produced on the skin following exposure to the ultraviolet radiation of the sun. This exposure leads to the conversion of 7‐dehydrocholesterol (7‐DHC) to pre‐vitamin D3 which isomerizes to vit D3.^11^ In the canonical pathway, D3 is activated by a 25‐hydroxylase (CYP2R1 or CYP27A1) and 1a‐hydroxylase (CYP27B1) to produce 1,25‐dihydroxyvitamin D3 (1,25(OH)2D3 which is the major hormonally active form of vit D.^12^ Vit D is also available exogenously from food sources or dietary supplements.^13^ It was primarily recognized for supporting bone health through calcium homeostasis, but recently, it has drawn attention to its critical role in modulating immune cells and inhibiting the inflammatory response.^14^ Previous studies have indicated a relationship between vit D deficiency and some cancers,^15^ certain autoimmune,^16^ and infectious viral diseases such as SARS, MERS, and Influenza A.^17^ In addition, there is evidence that vit D supplementation effectively decreases the level of proinflammatory cytokines such as TNF‐α and IL‐6, stimulates anti‐inflammatory cytokines such as IL‐10 and IL‐12,^3^ and reduces the rate of ICU admission of COVID‐19‐infected patients.^18^

One possible mechanism of the effect of vit D in COVID‐19 is related to angiotensin‐converting enzyme 2 (ACE2). This enzyme serves as the major entry point for SARS‐CoV‐2 into cells via binding a “spike” protein on its surface to ACE2 within the host.^19^ ACE2 is a key regulator for maintaining homeostasis and negatively regulates the renin‐angiotensin‐aldosterone system (RAAS) in humans. Chronic activation of RAAS is associated with exacerbating inflammation.^20^ By attaching SARS‐CoV‐2 to ACE2, the ACE/ACE2 balance is disrupted and RAAS activates leading to COVID‐19 progression.^21^

Vit D is essential for the normal development of antigen‐presenting cells, induction of macrophage expression, stimulation of the chemotaxis of neutrophils, monocytes, macrophages, and T cells, and clearance of respiratory pathogens through apoptosis.^22^

Prevalence of vit D deficiency among the Iranian population has been reported, with about half of the Iranian people reportedly having vit D deficiency.^23^ On the other hand, the ratio of deaths to total infected people with COVID‐19 is relatively much higher in Iran compared to most other countries^2^ which could mean that widespread Vit D deficiency predisposes individuals to COVID‐19.^24^ Based on evidence; patients supplemented with Vit D were associated with better and reduced severity of COVID‐19 outcomes.^25^, ^26^ Another point that some studies have mentioned is the time to start the treatment, the earlier the disease is better controlled.^27^ We should also consider that studies have shown that critically ill patients have a very high prevalence of vit D deficiency which is clearly associated with greater illness severity and mortality.^28^ According to all the mentioned factors above, it is imperative to investigate the role of vit D supplementation in COVID‐19 and explore its vital role in health particularly in the context of improved nutritional health and prevention of COVID‐19‐related complications. The aim of this case‐control study was to investigate the relationship between vit D supplementation and inflammatory, biochemical, and mortality indices in critically ill patients with COVID‐19.

## 2 METHODS

### 2.1 Participants

This case‐control study was performed on 200 critically ill patients with COVID‐19 aged 35−85 year, who were hospitalized in the ICU of Razi Hospital in Rasht, Iran during the summer and autumn of 2020. In total, 100 patients with a survival of more than 30 days as the case group and 100 patients with a survival of fewer than 30 days as the control group were included in the study. The inclusion criteria are consent for participation, diagnosis of COVID‐19 based on positive PCR results, CT scan confirmation of lung involvement, and hospitalization in the ICU at least for 1 week, and receive enteral nutritional support. The exclusion criteria were a history of lung and/or heart diseases and/or the presence of malignant tumors, the recent usage of chemotherapy drugs, vit D supplementation before hospitalization, and insufficient medical records.

The present study was approved by the ethical committee of Shahid Beheshti University of Medical Sciences, Tehran, Iran (code: IR.SBMU.CRC.REC.1399.031), and informed written consent was obtained from all participants before the study.

### 2.2 Patients' characteristics

The information of patients with COVID‐19 eligible to participate in the study was collected from the medical records and formal written consent was obtained from all the patients and/or relevant authorities as appropriate. Data on general, social, demographic, and pathological characteristics were collected from the medical records. All the participants were selected from the same city and the measurements were made in the same season. They had been homogenized in terms of the time interval between the first diagnosis and admission to the hospital and also received the same medical treatment. The patient's height was determined by using a height gauge and measuring the length of the patient's ulna,^29^ and weight was measured according to the arm's circumference^30^ and using standard charts.

### 2.3 Vit D intake

According to the opinion of some especialists and dietitians, some ICU patients received vit D3 supplements. Using the information from the ICU sheet, the patient's nutritional status and use of vit D supplements (drop of vit D3 10,000 IU, daily) were assessed. Dietary intake of vit D over the past year was assessed as a covariate and the food frequency questionnaire (FFQ) was used to obtain information on vit D intake over the past year. All participants were interviewed face‐to‐face by trained interviewers to obtain necessary information on a completed validated 148‐item semiquantitative FFQ with a standard serving size commonly consumed by Iranian people.^31^ The participants or their first degree relatives were asked how often they had consumed these foods during the last year. Some patients were conscious but could not get out of bed. In the case of nonconscious patients, necessary nutritional information was collected from first degree relatives.

The frequency of consumption of a given serving of each food item was collected on a daily (e.g., bread), weekly (e.g., meat), or monthly (e.g., fish) basis, and data were transformed into the average daily intakes. The portion sizes were then converted to grams by using household scales. Consumption of food items in grams was then calculated by multiplying the portion size by daily intake frequency. Vit D consumption from all dietary sources was computed by Nutritionist IV software. Since the Iranian food composition table (FCT) is not comprehensive, an analysis of energy and nutrients was done using the United States Department of Agriculture FCT. The vit D supplementation during admission to hospital was assessed using data recorded in hospital information systems. The vit D supplementation was evaluated based on the number of doses administered (10,000 IU/day vit D) to evaluate the association between vit D supplementation and pathological and biochemical indicators, as well as patient mortality.

### 2.4 Inflammatory and biochemical indices

We assessed some laboratory tests which are reported to provide critical information regarding prognosis, disease course, and response to therapy.^32^, ^33^ The level of hemoglobin (Hb), platelet (Plt), hematocrit (Hct), WBC, neutrophils, lymphocytes, glasgow coma scale (GCS), acute physiology and chronic health evaluation (APACHE), blood sugar (BS), lactate dehydrogenase (LDH), blood urea nitrogen (BUN), creatinine (Cr), albumin (Alb), sodium (Na), potassium (K), calcium (Ca), phosphorus (P), potential hydrogen (PH), oxygen saturation (O_2_ sat), partial pressure of oxygen (PO_2_), partial pressure of carbon dioxide (PCO_2_), bicarbonate (HCO_3_), base excess (Be), prothrombin time (PT), partial thromboplastin time (PTT), international normalized ratio of PT of blood coagulation (INR), IL, erythrocyte sedimentation rate (ESR), and mean arterial pressure (MAP) were collected from the patient's medical records. These measurements were routinely performed in the hospital during hospitalization by standard kits and are considered highly reliable.

### 2.5 Statistical analysis

The characteristics of demographic, social, and anthropometric indicators of the participants were described using descriptive statistics based on the mean and standard deviation (for quantitative data) and percentages and numbers (for qualitative data). SPSS version 20 was used for the statistical analysis. Due to multiple comparisons, significance thresholds were corrected using Bonferroni to reduce the probability of false positives and all biomedical and pathological comparison between the case and control groups were considered significant at *p* < .001 (Tables 2 and 3). Finally, logistic regression method with odds ratio (ORs) and 95% confidence intervals (CIs) was used to investigate the association between 30 days survival and vit D supplementation. We analyzed this relationship in three different models: model 1: crude, model 2: adjusted for age and sex (as the effective factors on the vit D requirements^34^ and COVID‐19 severity^35^), and model 3: adjusted for underlying diseases and smoking. *p* < .05 was considered significant.

## 3 RESULTS

The normal distribution of the data was confirmed using the Kolmgrove−Smirnov method. The general characteristics of the participants are presented in Table 1. Grouping of the participants was performed based on survival for >30 days versus dead. The patients with a survival for more than 30 days had higher vit D3 supplementation (drop of vit D3 10,000 IU, daily) duration compared to the died patients (9 ± 4.4 vs. 3.3 ± 1.9 day, *p* = .001). No significant differences were observed for BMI, daily formula intake, age, sex, weight, height, APACHE II scores, and dietary vit D intake between the groups.

**Table 1:** Characteristics of the participants.

Measurements	Survival more than 30 day (*n* = 100)	Dead within 30 day (*n* = 100)	*p* Value
Body mass index (kg/m^2^)	26.5 ± 3	27.7 ± 5.2	.20
Daily formula intake (cc/day)	361 ± 292.9	247 ± 252.2	.14
Age (year)	57 ± 14.9	64 ± 14.7	.10
Gender			
Males	17%	83%	.71
Female	20.0%	80.0%	.71
Body weight (kg)	74 ± 11.1	76 ± 13.5	.43
Height (cm)	167 ± 7.7	166 ± 6.5	.64
APACHE II	15 ± 1.5	16 ± 2	.18
ICU days	15 ± 7.1	4 ± 2.1	<.001
Vit D supplementation duration (day)	9 ± 4.4	3.3 ± 1.9	.001
Dietary intake of vitamin D (μg/day)	1.45 ± 1.17	1.34 ± 1.07	.53

Abbreviations: APACHE II, acute physiology and chronic health evaluation; ICU, intensive care unit; Vit D, vitamin D.

Regarding biochemical and pathological assessments, the participants in the survived group had a lower eosinophile (2.2 ± 0.5 vs. 6 ± 0.0, *p* < .001) compared to those who were in the dead group (Table 2). Between these two groups, no significant differences in BS, Na, K, BUN, Cr, Alb, HCT, Ca, P, MAP, O_2_ sat, PO_2_, PCO_2_, arterial PH, HCO_3_, Be, WBC, neutrophil, lymphocyte, monocyte, GCS, Hb, Plt, PT, PTT, INR, ESR, defection, urine volume, daily formula were evident.

**Table 2:** Biochemical and pathological indices among the participants based on survival of the patients.

Measurements	Survival > 30 day (*n* = 100)	Dead within 30 day (*n* = 100)	*p* Value^a^
BS (mg/dL)	136 ± 39	144 ± 39	.44
Na (mEq/L)	139 ± 5.2	141 ± 7.9	.41
K (mEq/L)	4 ± 0.4	4.1 ± 0.4	.60
BUN (mg/mL)	35 ± 15.7	42 ± 18.4	.15
Cr (mg/mL)	1.5 ± 0.9	1.6 ± 1.1	.59
Alb (g/dL)	2.9 ± 0.3	3.1 ± 1.2	.52
HCT (%)	29 ± 4.3	30 ± 2.5	.17
Ca (mg/dL)	7.9 ± 0.4	7.8 ± 0.4	.85
P (mg/dL)	3.2 ± 0.9	3.2 ± 0.1	.82
MAP	72 ± 9.4	71 ± 6.7	.54
O_2_ sat	81 ± 10.2	77 ± 8.1	.18
Arterial PH	7.29 ± 0.07	7.26 ± 0.05	.01
PO_2_ (mmHg)	60 ± 18.03	65 ± 16.3	.29
PCO_2_ (mmHg)	46 ± 12.9	43 ± 6.1	.35
HCO_3_ (mEq/L)	22 ± 8.4	18 ± 3	.08
Be (mEq/L)	−3.1 ± 7.4	−6.9 ± 4.1	.12
WBC (10^6^/L)	13.2 ± 5.4	13.5 ± 2.8	.85
Neotrophil (10^6^/L)	87 ± 4.6	87 ± 2.7	.66
Lymphocyte (10^6^/L)	11.7 ± 3.6	11.6 ± 2.7	.91
Monocyte (10^6^/L)	2.2 ± 1.2	1.6 ± 0.5	.16
Eosinophile (10^6^/L)	2.2 ± 0.5	6 ± 0.0	<.001
GCS	8 ± 2	8 ± 1.5	.59
Hb (g/dL)	9.2 ± 1.5	9.8 ± 1.05	.17
Plt	177 ± 98.2	166 ± 64	.65
PT (s)	14 ± 2.3	12 ± 0.6	.02
PTT (s)	42 ± 8.4	43 ± 11	.63
INR	1.1 ± 0.2	1.03 ± 0.1	.16
ESR (mm/h)	89 ± 5.2	88 ± 5.0	.90
Defecation	1 ± 0.00	1 ± 0.00	<.001
Urine volume (mL)	2169 ± 948.9	18844 ± 894	.22
Daily formula	361 ± 292.9	247 ± 252.2	.14

Abbreviations: Alb, albumin; Be, base excess; BS, blood sugar; BUN, blood urea nitrogen; Ca, calcium; Cr, creatinine; ESR, erythrocyte sedimentation rate; GCS, glasgow coma scale; Hb, hemoglobin; HCO_3_, bicarbonate; Hct, hematocrit; INR, international normalized ratio of prothrombin time of blood coagulation; K, potassium; MAP, mean arterial pressure; Na, sodium; O_2_ sat, oxygen saturation; P, phosphorus; PCO_2_, partial pressure of carbon dioxide; pH, potential hydrogen; Plt, platelet; PO_2_, partial pressure of oxygen; PT, prothrombin time (test); PTT, partial thromboplastin time (test); WBC, white blood cell.

^a^ Assessed by independent *t*‐test.

The distribution of biochemical and pathological indices based on vit D supplementation is shown in Table 3. No significant differences were found between the two groups in BS, Na, K, BUN, Cr, Alb, HCT, Ca, P, MAP, arterial PH, O_2_ sat, PO_2_, PCO_2_, HCO_3_, Be, WBC, neutrophil, lymphocyte, eosinophile, monocyte, GCS, Hb, Plt, PT, PTT, INR, IL6, Pct, ESR, defection, urine volume, daily formula.

**Table 3:** Biochemical and pathological indices among the participants based on vitamin D supplementation.

**Measurements**	**With vitamin D supplementation (*n*** = **62)**	**Without vitamin D supplementation (*n*** = **138)**	***p*** **Value** a
BS (mg/dL)	143 ± 48.8	144 ± 34.9	.96
Na (mEq/L)	139 ± 5	141 ± 8.2	.27
K (mEq/L)	4 ± 0.4	4.1 ± 0.4	.14
BUN (mg/mL)	35 ± 19.7	43 ± 16.9	.07
Cr (mg/mL)	1.4 ± 0.9	1.7 ± 1.1	.24
Alb (g/dL)	2.9 ± 0.3	3.1 ± 1.05	.35
HCT (%)	30 ± 3.6	30 ± 2.8	.67
Ca (mg/dL)	8 ± 0.4	8 ± 0.3	.79
P (mg/dL)	3.3 ± 1.2	3.1 ± 0.7	.51
MAP	74 ± 10.1	70 ± 6.5	.06
O_2_ sat	82 ± 8.2	76 ± 8.2	.003
Arterial PH	7.3 ± 0.07	7.3 ± 0.05	.20
PO_2_ (mmHg)	66 ± 23.4	63 ± 12.5	.48
PCO_2_ (mmHg)	44 ± 11.3	44 ± 7.2	.98
HCO_3_ (mEq/L)	21 ± 7.9	18 ± 4	.13
Be (mEq/L)	−5.3 ± 7.7	−6.5 ± 3.8	.44
WBC (10^6^/L)	18 ± 29	14 ± 3.3	.53
Neotrophil (10^6^/L)	87 ± 4.93	8 ± 2.1	.70
Lymphocyte (10^6^/L)	11 ± 4.13	12 ± 2	.70
Monocyte (10^6^/L)	1.7 ± 0.67	2.2 ± 1.3	.28
Eosinophile (10^6^/L)	2.8 ± 1.79	3.0 ± 0	.01
GCS	8 ± 1	8 ± 1.8	.92
Hb (g/dL)	9 ± 1.3	10 ± 1.1	.30
Plt	177 ± 88.2	167 ± 66.7	.64
PT (s)	13 ± 1.8	12 ± 1	.19
PTT (s)	42 ± 8.4	43 ± 11.3	.67
INR	1.07 ± 0.2	1.03 ± 0.1	.30
ESR (mm/h)	90 ± 3.00	88 ± 5.1	.43
Urine volume (mL)	2044 ± 822.4	1864 ± 943.8	.38
Daily formula	315 ± 294.3	246 ± 242.4	.28

Abbreviations: Alb, albumin; Be, base excess; BS, blood sugar; BUN, blood urea nitrogen; Ca, calcium; Cr, creatinine; ESR, erythrocyte sedimentation rate; GCS, glasgow coma scale; Hb, hemoglobin; HCO_3_, bicarbonate; Hct, hematocrit; INR, international normalized ratio of prothrombin time of blood coagulation; K, potassium; MAP, mean arterial pressure; Na, sodium; O_2_ sat, oxygen saturation; P, phosphorus; PCO_2_, partial pressure of carbon dioxide; pH, potential hydrogen; Plt, platelet; PO_2_, partial pressure of oxygen; PT, prothrombin time (test); PTT, partial thromboplastin time (test); WBC, white blood cell.

^a^ Assessed by independent *t*‐test.

Raw and adjusted logistic regression was used to explore the relationship between the survival >30 days and vit D supplementation among participants (Table 4). Vit D supplementation was positively associated with the survival time (OR: 1.98, 95% CI: 1.15−3.40, *p* = .013). The association remained significant after adjusting for age and sex (model 2) and additional adjustments for underlying diseases and smoking (model 3).

**Table 4:** The association between 30 days survival and vitamin D supplement intake.

	**Model 1**		**Model 2**		**Model 3**	
	**OR (95% CI)**	***p***	**OR (95% CI)**	***p***	**OR (95% CI)**	***p***
Vitamin D	1.98 (1.15−3.40)	.013	2.38 (1.14−4.97)	.021	3.21(1.05−9.77)	.04

*Note*: Model 1: crude, Model 2: adjusted for age and sex, Model 3: further adjustments for underlying diseases and smoking.

## 4 DISCUSSION

This case‐control study investigated the relationship between vit D supplementation and inflammatory, biochemical, and mortality indices in critically ill patients with COVID‐19. A total of 200 critically ill COVID‐19 patients were grouped into those who survived for >30 days and those who died within 30 days. It was evident that the participants receiving vit D supplementation had lower eosinophile level in comparison with participants without vit D supplementation. Results from a cross‐sectional study on 669 men and women showed that vit D deficiency was related to higher blood eosinophil count supporting the possible role of vit D in the eosinophil immune response.^36^ Furthermore, a review study on vit D high doses as an alternative to prevent COVID‐19 infection reported an positive association between vit D supplementation and suppressing the recruitment of eosinophils and lymphocytes in the airways and decreasing inflammatory response.^37^

In the present study, no significant alteration was observed in other hematological, biochemical, and inflammatory parameters, which is in line with several previous studies.^38^, ^39^, ^40^ A double‐blind, randomized, placebo‐controlled trial by Khorasanchi et al. also evaluated the effect of vit D supplementation on biochemical and inflammatory factors of hospitalized COVID‐19 patients^41^ and reductions in the levels of CRP and LDH were reported in the COVID‐19 patients supplemented with vit D as compared with the control group. They also reported that there was no change in several other biochemical and inflammatory factors such as Triglyceride, total cholesterol, fasting plasma glucose, alanine aminotransferase, aspartate aminotransferase, alkaline phosphatase, BUN, Cr, Ca, P, and Alb. In contrast to the findings of this study on the lack of change in the levels of inflammatory markers, several other studies^42^, ^43^, ^44^ reported a positive relationship between reduction in some inflammatory markers (e.g., IL6, CRP, fibrinogen) and vit D supplementation. The fact that many inflammatory and hematological parameters have a longer half‐life, hence they require a longer time frame to reflect the effect of vit D supplementation. The short‐term follow‐up may be an underlying reason why we and others didn't find a difference in several biochemical and inflammatory markers evaluated. Thus, it is imperative to conduct an extended follow‐up recording of these markers so as to better examine the paraclinical results.

The most conspicuous finding of the present study was that vit D supplementation is positively associated with survival duration. This finding is consistent with the results of several systematic reviews and meta‐analyses, that assessed the impact of vit D supplementation and mortality in patients hospitalized with COVID‐19 and noted that vit D supplement was related to a reduction in the risk of COVID‐19 mortality.^42^, ^45^, ^46^, ^47^, ^48^, ^49^, ^50^, ^51^, ^52^

Vit D can be activated through canonical pathways in which 1,25(OH)2D3 was produced by CYP2R1 and CYP27A1 in the liver and then CYP27B1 in the kidney. In noncanonical pathways, 20(OH)D3 and 20,23(OH)2D3 are produced in humans by CYP11A1.^53^ There are strong experimental studies reported the classical 1,25(OH)2D3 and novel CYP11A1‐derived hydroxyderivatives have immunomodulatory and anti‐inflammatory effects that can reduce the cytokine storm by enhancing the innate immune response and modulating the acquired immune system response to COVID‐19.^53^, ^54^, ^55^, ^56^ Several recent studies revealed that vit D can prevent viral replication and cytokine storm simultaneously^57^, ^58^, ^59^, ^60^, ^61^ and thereby contribute to lower mortality in critically ill COVID‐19 patients as evident in the current study.

In a recent clinical trial with 50 hospitalized COVID‐19 patients, De Niet et al. reported that vit D, either 25,000 IU every day for 4 days or 25,000 IU every week for 6 weeks reduced the hospitalization and intubation time.^62^ Fiore et al. in a monocentric matched‐cohort study with 58 COVID‐19 patients who received 100,000 UI/daily vit D3 for 2 days also reported that vit D supplementation was significantly associated with the survival rate.^63^ The study done on 76 Spanish patients suggests that the administration of a high dose of calcifediol or 25‐hydroxyvitamin D3 (on the day of admission, Day 3, Day 7, and then weekly until discharge or ICU admission) significantly reduced the need for ICU treatment of Covid‐19 patients. So that, Of 50 patients treated with calcifediol, 2% required admission to the ICU, while this ratio was 50% in untreated patients.^64^ While there is a clear evidence of the positive effect of vit D supplementation in the survivality of COVID‐19 patients, some other studies could not find such an effect and noted that vit D supplementation had no benefit for mortality in COVID‐19.^65^, ^66^, ^67^

like Castilo et al. study, serum 25OHD concentrations at baseline or during treatment are not available.^64^ Overall, previous studies have repeatedly shown that more than 50% of adults living in Iran^68^ are vit D deficient (25.41 ng/mL on average).^69^ On the other hand, this deficiency has been clearly observed in hospitalized patients, which is associated with greater disease severity.^70^

There are many possible mechanisms introduced to elaborate the relationship between COVID‐19 and vit D.^71^, ^72^, ^73^ One mechanism may be related to creating a defense against the virus partly via induction of cathelicidin (LL‐37) and defensins. LL‐37 has a role in many steps of viral infection and it can affect non‐enveloped and enveloped viruses.^74^ In addition, a higher level of LL‐37 in serum leads to lower expression of IL‐17. The available data indicates that IL‐17 takes part in the pathology of COVID‐19 such as in thrombosis^75^ and ARDS.^76^ Therefore, upregulating IL‐17 my have a role in the relationship between hypovitaminosis D with the severe complications and severity of COVID‐19. Another mechanism that connects COVID‐19 and vit D is regulating inflammatory cytokines production. Vit D upregulates anti‐inflammatory cytokines like IL‐19 and downregulates proinflammatory cytokines like TNF‐a, IL‐6, and IL‐21. This change from a proinflammatory to an anti‐inflammatory state may decrease the risk of cytokine storm in COVID‐19.^77^

Another mechanism through which vit D is beneficial in COVID‐19 is through modulation of the activity of the RAAS and ACE2. As Hill et al. pointed out in their study, vita D plays a controlling role in the ACE/angiotensin (Ang) II/angiotensin type I receptor axis and the ACE2/Ang axis activity, which is used by SARS‐CoV‐2 to attack host cells.^78^ This increases the concentration and expression of ACE2 and Ang. A key anti‐inflammatory and antioxidant role is played by ACE2/Ang that protects the lung against ARDS. In fact, ACE2 plays a protective role against avian influenza A H5N11.^79^, ^80^ Therefore, upregulation of the ACE2/Ang can be considered a potential way to protect against ARDS and acute lung injury.^73^, ^81^ A novel mechanism has also been proposed for the role of active forms of vit D and lumisterol (L3) in the inhibition of SARS‐CoV‐2 replication machinery enzymes. The main protease (Mpro) and RNA‐dependent RNA polymerase (RdRP) play important roles in SARS‐CoV‐2 replication. With a high UVB exposure, pre‐D3 undergoes photoisomerization to L3. Based on the reported mechanism, vit D3, and L3 hydroxyderivatives indicate a complementarity with SARS‐CoV‐2 Mpro and RdRP binding pockets, therefore, inhibiting the enzymatic activity.^11^

Vit D supplementation in critically ill patients with COVID‐19 can potentially lead to higher survivability. However, the present study had some limitations, First of all, because of the case‐control design of the study, it was not possible to estimate the cause‐and‐effect relationship. Second, the duration of the study was short while the manifestation of the effect of inflammatory and hematological parameters may require a longer time. Third limitation related to the low number of participants under investigation. Fourth, assessment of serum vit D was not part of the hospital's routine measurements. So, the serum vit D level was not available. Future randomized clinical trials with larger groups of participants and more comprehensive measurements are recommended. Further observational and experimental studies are required to investigate the underlying mechanism of how exactly vit D supplementation can influence the survival of COVID‐19 patients. Finally, considering that this study was conducted on adults (35−85 years old), these results may need consideration before being generalizable to younger adolescents and children. In spite of the limitations, it is believed that the present study gave valuable insights into the potential role of vit D supplementation for COVID‐19 management.

## 5 CONCLUSION

In conclusion, vit D supplementation in critically ill patients with COVID‐19 can significantly decrease the mortality rate. However, no significant difference was found in the biochemical and pathological indices except eosinophile level. Further studies including randomized clinical trials might be useful for providing the underlying mechanism of vit D‐mediated beneficial effect on COVID‐19.

## AUTHOR CONTRIBUTIONS

*Conception and design*: Maryam Gholamalizadeh, Saeid Doaei, and Mokammad Esmail Akbari. *Acquisition of data*: Faezeh Rabbani, Mina Ahmadzadeh, and Azadeh Hajipour. *Analysis and interpretation of data*: Hayehe Musavi, Khadijeh Abbasi Mobarakeh, Zahra Salimibeni, and Bojlul Bahar. *Study supervision*: Zahra Mahmoodi, Somayeh Gholami, and Samaneh Mirzaei Dahka. *Writing, review, and/or revision of the manuscript*: All authors.

## CONFLICT OF INTEREST STATEMENT

The authors declare no conflict of interest.

## ETHICS STATEMENT

This study was approved by the Institutional Review Board at Shahid‐Beheshti University of Medical Sciences (code: IR.SBMU.CRC.REC.1399.031). All patients signed an informed consent form at baseline. Institutional consent forms were used in this study.
